# 2568 Pembrolizumab for patients with leptomeningeal disease from advanced solid tumors

**DOI:** 10.1017/cts.2018.174

**Published:** 2018-11-21

**Authors:** Jarushka Naidoo, Chen Hu, Karisa Schrek, Roisin Connolly, Cesar Santa-Maria, Evan Lipson, Ranee Mehra, Bettegowda, Kristin Redmond, Arun Venkatesan, Stuart Grossman

**Affiliations:** Johns Hopkins University School of Medicine

## Abstract

OBJECTIVES/SPECIFIC AIMS: Pembrolizumab is an anti-PD-1 immune checkpoint antibody that has demonstrated promising anti-tumor activity in patients with solid tumor malignancies, including patients with brain metastases from malignant melanoma and non-small cell lung cancer. Leptomeningeal disease (LMD) is a rare form of malignant spread to the central nervous system (CNS), that occurs in 2%–10% of patients with solid tumors, most commonly in breast cancer and non-small cell lung cancer. We propose an open-label phase II study of pembrolizumab in patients with LMD from advanced solid tumors (NCT03091478). This study aims to determine if pembrolizumab therapy can lead to a radiologic, cytologic or clinical response in the CNS, in patients with LMD. METHODS/STUDY POPULATION: Patients with pathologically confirmed advanced solid tumors, and either radiologic or cytologic evidence of LMD, will be identified at a single institution. Radiologic LMD will be defined as a >4 mm area of measurable LMD on gadolinium-enhanced MRI brain/total-spine; and cytologic LMD will be defined as the presence of malignant cells on CSF cytology. Patients will be excluded if they have: active autoimmune conditions that require immunosuppression, received radiation therapy to the only area of measurable LMD within 3 months of study enrollment, have an ECOG performance status <1. Once enrolled, patients will receive pembrolizumab 200 mg intravenously every 3-weeks, until disease progression or unacceptable toxicity. Patients will have CSF sample sampling, blood draws, radiologic imaging of the body (CT), brain/total-spine (gadolinium-enhanced MRI) pre-treatment, after 2 and after 4 cycles of therapy, for response assessment and correlative studies. The primary endpoint of the study is CNS response assessed at 12 weeks/after 4 cycles of pembrolizumab, defined either as radiologic response (reduction in size of LMD on gadolinium-enhanced MRI) and/or cytologic response (conversion of positive to negative CSF cytology on 2 consecutive samples) and/or clinical response. Secondary endpoints will include progression-free survival, overall survival, and safety. To explore the mechanisms by which pembrolizumab may affect LMD, we will assess dynamic changes in genomic and immunologic markers in the CSF and serum pre and post pembrolizumab using next-generation sequencing and multi-color flow cytometry, respectively. RESULTS/ANTICIPATED RESULTS: We will aim to accrue a total of 20 patients, allowing for a 10% drop-out rate, the final sample size will include 18 patients who have received at least 1 dose of pembrolizumab. CNS-response at 12 weeks will be assessed radiologically +/− cytologically, and the proportion of patients with CNS response and associated 95% confidence interval with be reported. CNS-progression-free survival and overall survival will be assessed using the Kaplan-Meier method. Cause of death will be recorded. Safety will be assessed as detailed above, and monitored as per an institutional Data Safety and Monitoring Plan. Exploratory endpoints will include genomic testing of tumor cells and cell-free DNA in CSF and serum, and immunologic studies of immune cells in CSF and serum at pre-defined timepoints. These data will be presented descriptively. We conservatively estimate that we will accrue 1 patient per month at our institution. Study duration will be approximately 24 months, allowing 18 months for accrual and 6 months for follow-up and data analysis. DISCUSSION/SIGNIFICANCE OF IMPACT: There are no currently FDA-approved therapies for patients with LMD from solid tumors. Anti-PD-1 immunotherapy is a promising class of agents, with known efficacy in patients with CNS metastatic disease, across tumor types. This study seeks to identify whether pembrolizumab may lead to CNS responses in patients with LMD. Additionally, genomic and immunologic analyses in CSF and blood pre and post-pembrolizumab may identify mechanisms by which immunotherapy affects the CNS in patients with LMD.

